# High-Speed Die Bond Quality Detection Using Lightweight Architecture DSGβSI-SECS-Yolov7-Tiny

**DOI:** 10.3390/s25237358

**Published:** 2025-12-03

**Authors:** Bao Rong Chang, Hsiu-Fen Tsai, Wei-Shun Chang

**Affiliations:** 1 Department of Computer Science and Information Engineering, National University of Kaohsiung, Kaohsiung 81148, Taiwan; brchang@nuk.edu.tw (B.R.C.); m1125513@mail.nuk.edu.tw (W.-S.C.); 2 Department of Fragrance and Cosmetic Science, Kaohsiung Medical University, Kaohsiung 80708, Taiwan

**Keywords:** depthwise separable convolution, ghost convolution, SE layer, ECA net, coordinate attention, small object enhancer, DSGβSI-SECS-Yolov7-tiny

## Abstract

The die bonding process significantly impacts the yield and quality of IC packaging, and its quality detection is also a critical image sensing technology. With the advancement of machine automation and increased operating speeds, the misclassification rate in die bond image inspection has also risen. Therefore, this study develops a high-speed intelligent vision inspection model that slightly improves classification accuracy and adapts to the operation of new-generation machines. Furthermore, by identifying the causes of die bonding defects, key process parameters can be adjusted in real time during production, thereby improving the yield of the die bonding process and substantially reducing manufacturing cost losses. Previously, we proposed a lightweight model named DSGβSI-YOLOv7-tiny, which integrates depthwise separable convolution, Ghost convolution, and a Sigmoid activation function with a learnable β parameter. This model enables real-time and efficient detection and prediction of die bond quality through image sensing. We further enhanced the previous model by incorporating an SE layer, ECA-Net, Coordinate Attention, and a Small Object Enhancer to accommodate the faster operation of new machines. This improvement resulted in a more lightweight architecture named DSGβSI-SECS-YOLOv7-tiny. Compared with the previous model, the proposed model achieves an increased inference speed of 294.1 FPS and a Precision of 99.1%.

## 1. Introduction

**Figure A1:**
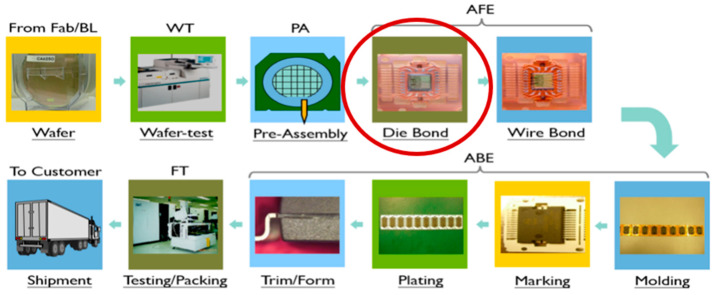
IC packaging and testing process.

In the IC packaging and testing process, one of the essential steps is the die bonding process, and it uses epoxy adhesive or solder to attach the die to the lead frame [1]. The die bonding process requires real-time inspection and prediction of its yield, and timely adjustment of machine operating parameters can effectively reduce manufacturing cost losses [2]. Figure A1 illustrates the complete IC packaging and testing process, from wafer thinning to the key steps leading up to final shipment [3]. For the die bonding process, the epoxy resin or solder is dispensed onto the die pad area of the lead frame in a specified pattern, usually in a star shape. The die is removed from the carrier tape and placed onto the dispensed epoxy resin through the pick-and-place process. Die bonding technology is a crucial stage in the front-end semiconductor packaging process that affects the overall yield.

This study acquired image data using a high-resolution image-sensing camera from a well-known semiconductor manufacturer in southern Taiwan to obtain training and testing datasets. We manually labeled the sensed images and analyzed the bonding conditions of each die’s four sides and corners. In other words, this study used this task to classify images into two categories: bond_good (including side_good and corner_good), indicating that the die is correctly attached to the IC substrate that means epoxy resin coating extends beyond the side or corner boundary; and bond_bad (including side_bad and corner_bad), indicating that the die is not correctly attached to the IC substrate that means epoxy resin coating might not extend beyond the side or corner boundary. Finally, this study uses a two-bit hexadecimal code to represent the die bond result, allowing the classification between bond_good and bond_bad categories. Applying this coding method can quickly identify the situation of each die bond. This examination can determine which side or corner of the die doesn’t correctly attach to the IC substrate.

This study aims to improve the previously proposed DSGβSI-YOLOv7-tiny [3]. With the advancement of automated equipment in production lines, a model with faster inference speed is required to achieve real-time detection and online parameter adjustment. First, we did not adopt the latest version of the YOLOv11n model [4] because its number of parameters is four times larger than the proposed model in this study. The model has many parameters, which may not be suitable for typical embedded systems. Furthermore, during the implementation of the lightweight prediction model, the previously proposed DSGβSI-YOLOv7-tiny architecture showed insufficient computational complexity, resulting in a slight decrease in detection accuracy. Therefore, in addition to designing a more lightweight model, we attempted to modify some structures by introducing modules that can enhance target features to improve detection and prediction accuracy and ensure the effectiveness of the die bond process. This study also refers to related methods of the improved YOLOv7-tiny [5,6], exploring their improvements in convolutional computation and the application of various target enhancement modules for lightweight architectures used in fast wafer surface inspection. At the same time, we also refer to the application of YOLOv7-VD in intelligent vehicle vision detection [7] and the practical experience of automatic optical inspection (AOI) and recognition technology in die bond [8].

In the method design, this study proposes several models for performance comparison, including CGSE-YOLOv7-tiny, Mobile-YOLOv7-tiny, ReG-YOLOv7-tiny, DSGReG-YOLOv7-tiny, DSGβSI-YOLOv7-tiny, and DSGβSI-SE-YOLOv7-tiny. Finally, this study selected the model with the best performance, DSGβSI-SECS-YOLOv7-tiny, and converted this trained model into TensorFlow Lite for further integration into the die bond machine control system. The main contribution of this study is to propose an image sensing and object detection system that outperforms previously developed models, effectively identifying the classes of die bond defects (bond_bad). The proposed approach assists engineers in promptly adjusting key machine parameters to reduce the occurrence of electrical failures and resistance abnormalities, improve the die bonding process yield, and significantly reduce manufacturing cost losses.

## 2. Related Works

### 2.1. Literature Review

Due to the limited hardware resources of embedded platforms, such as insufficient computational power, restricted memory capacity, and power consumption constraints, applications designed initially to perform die bond detection and recognition on high-performance GPU servers cannot achieve their expected performance in embedded environments. Accelerating die bond inference speed through model lightweighting techniques under limited hardware resources, while maintaining existing accuracy, has become an important research topic to ensure that the model can complete tasks effectively and efficiently.

Boustedt et al. [9] discussed methods for improving chip interconnection in die bonding to enhance yield. The flip-chip method is the most reliable chip interconnection technique, capable of achieving incredibly high yield at very low cost. The bumps on the chip, the chip carrier, and the interconnection method between the chip and the carrier are the fundamental elements that constitute flip-chip interconnection. These elements are interdependent; therefore, we must comprehensively consider each component when selecting the optimal flip-chip system for a specific application.

On the other hand, Liao et al. [10] mentioned that as electronic devices advance, they become thinner, smaller, faster, and characterized by higher I/O density. Therefore, the bump diameter and pitch design will gradually become smaller or narrower to meet bump density requirements. This way will bring more challenges to semiconductor packaging processes, such as non-wetting and bridging issues during the die bonding process. Furthermore, X/Y/Z-axis bump misalignment and warpage mismatch can affect the assembly process of the Flip Chip Chip Scale Package (FCCSP).

Tsao et al. [11] further explained that during the chip mounting process, residual stress is generated within the die attach assembly, and material components experience stress due to the incompatibility of their coefficients of thermal expansion (CTE), resulting in out-of-plane displacement of the chip caused by the die attach process.

Gu et al. [12] proposed an improved YOLOv7-tiny architecture to meet edge devices’ real-time object detection requirements. They replaced the standard convolution in the ELAN structure with depthwise separable convolution (DWConv) to reduce the number of model parameters. To improve the neck network, they integrated the Coordinate Attention (CA) mechanism into the convolution to establish the Coordinate Attention Convolution (CAConv), which replaced the standard convolution. This approach demonstrated the feasibility and practicality of structural optimization for embedded platform applications.

In addition to depthwise separable convolution, introducing the Ghost module has become an essential technique for model lightweighting. Wang et al. [13] first integrated the Ghost module into the YOLOv7-tiny architecture. Following this, they introduced the CA module into the feature extraction network to enhance the model’s ability to learn defect location features. Then, they adopted the lightweight convolution module—Ghost Shuffle Convolution (GSConv)—in the feature fusion network, effectively reducing model parameters while maintaining satisfactory detection accuracy.

In the study by Gong et al. [14], GhostNet was combined with Dynamic Region Convolution (DRConv) and a cross-layer feature sharing network (CotNet Transformer) to enhance YOLOv7-tiny’s feature extraction and fusion capabilities. Implementing the Gaussian Error Linear Unit (GELU) to replace the conventional ReLU activation function improved the model’s nonlinear representation and classification performance. The enhanced model outperformed YOLOv7-tiny in accuracy, inference speed, and model size.

Tang et al. [15] also demonstrated the potential of combining the Ghost module with novel activation functions. The model improvements involved several key modifications: integrating the GhostNet V2 module into the YOLOv7-tiny backbone for parameter reduction; replacing LeakyReLU with FReLU in convolutional layers to enhance spatial feature modeling; and incorporating the SimAM, C3, and ODConv attention/convolution modules. Experimental results showed that the number of floating-point operations (FLOPs) decreased by 59%, the mAP dropped by only 0.64%, and the inference speed reached 47 fps, demonstrating outstanding performance improvement and application potential.

In summary, effectively lightweighting detection and recognition models by integrating techniques such as depthwise separable convolution, Ghost modules, attention mechanisms, and novel activation functions has become a significant research direction in edge computing for embedded deep learning models.

### 2.2. Data Collection and Preprocessing

**Figure A2:**
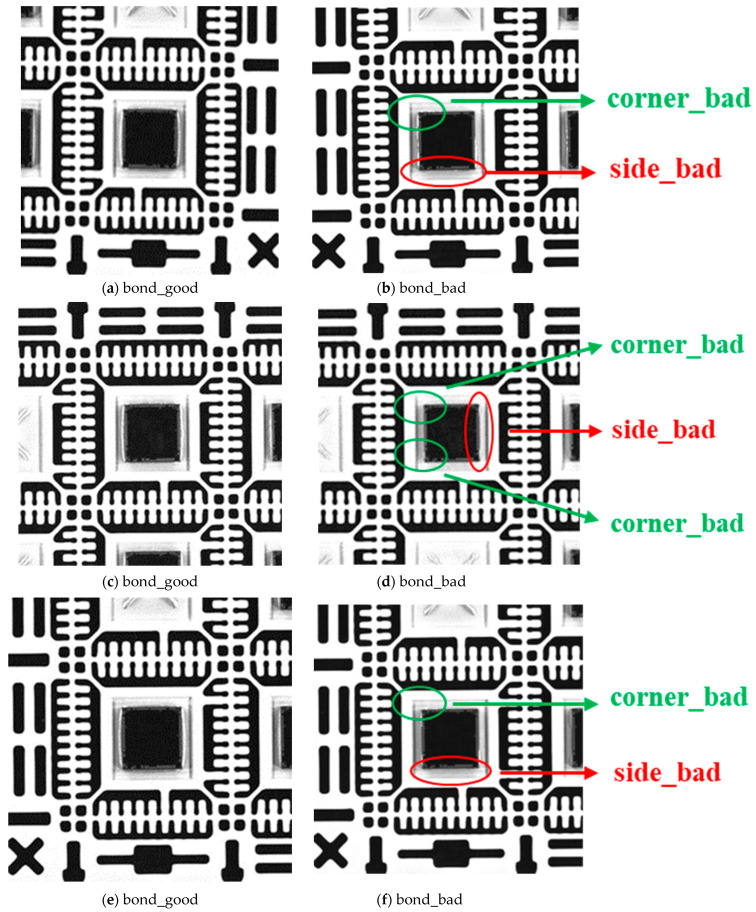
Sample images of die bond categories.

Based on our previous publication [3], the image data utilized in this study originated from a major semiconductor manufacturer in southern Taiwan. Figure A2 displays these images, all taken from a top-down perspective of the machine. The research team manually annotated these images for object detection and classification, defining four categories for the die’s bonding status: side_good, side_bad, corner_good, and corner_bad. Initially saved in XML format, the resulting label information required conversion to the YOLO label format to be compatible with YOLO-based model input.

For data collection, this study acquired 3145 images and split them into a training set (70%, 2204 images), a validation set (15%, 467 images), and a test set (15%, 474 images). This 70%:15%:15% split provides sufficient data support for all phases of model development.

To ensure effective learning of the complete die bonding status and accurate classification of side and corner quality, four model variations—CGSE-YOLOv7-tiny, Mobile-YOLOv7-tiny, ReG-YOLOv7-tiny, and DSGReG-YOLOv7-tiny—were initially trained. The experiment set hyperparameters as follows to optimize performance: 300 epochs (complete iterations over the dataset); a batch size of 32 (images per weight update); and an input image size of 1024 × 1024 to facilitate both computational efficiency and high accuracy in die bond object detection.

After training and validating the above models, this study further applied lightweight strategies to the DSGβSI-YOLOv7-tiny, DSGβSI-SE-YOLOv7-tiny, and DSGβSI-SECS-YOLOv7-tiny versions. These models integrate DSG convolution, ModifiedSiLU, AdaptiveSiLU activation functions, and attention mechanisms in their structure, aiming to significantly improve inference speed and computational efficiency while maintaining detection accuracy, thereby achieving a lightweight design suitable for embedded platform deployment. Through a systematic training process and data partitioning, this study ensures that the models deliver stable and reliable predictive performance across different datasets, providing an efficient and precise intelligent solution for die bond inspection.

### 2.3. Identifying Die Bond Pros and Cons

Drawing from our previous work [3], we input the 474 images of the test set into the fully trained model to generate predictions. The recognition result for every image consisted of the predicted class (specifying the bonding status, e.g., side_good or corner_bad) and its corresponding confidence level (the model’s certainty).

**Figure A3:**
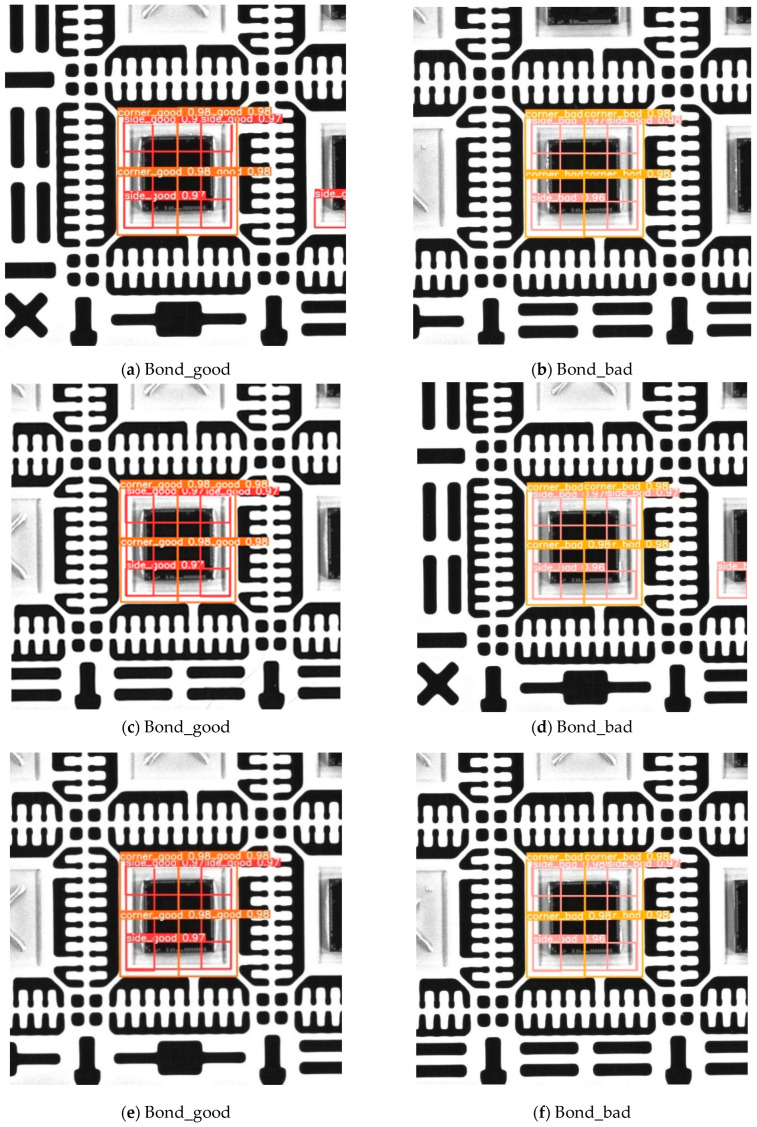
Die bond recognition.

Experimental results showed that all parts of the images achieved confidence levels of at least 95%, demonstrating the high stability and reliability of the model. When all four sides of a die met the complete bonding condition, the model predicted side_good; otherwise, it predicted side_bad. The same logic applied to the four corners: if the bonding condition was full, the prediction was corner_good, otherwise corner_bad, as shown in Figure A3. These results validate the proposed model’s ability to accurately recognize the bonding status of die sides and corners in die bond inspection tasks.

### 2.4. Detection of Die Bond Types

**Figure A4:**
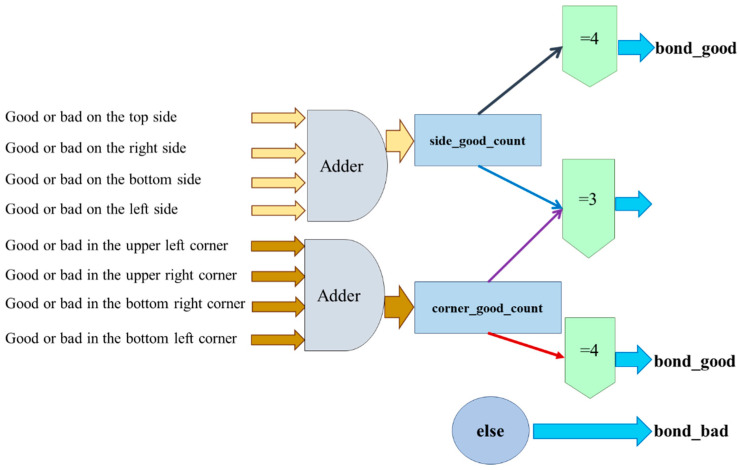
Judgment of categories with bond_good or bond_bad.

**Figure A5:**
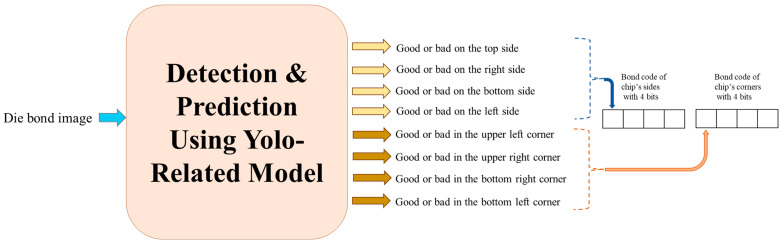
Any combination of the bond detection of the die’s sides and corners.

As detailed in our previous work [3], Figure A4 illustrates the four classification categories for die bond detection results (side_good, side_bad, corner_good, and corner_bad), with the classification rules provided by the manufacturer. Using this clas-sification standard, the model can effectively quantify the bonding completeness of die bonds, serving as an essential reference for subsequent analysis or adjustments of pro-duction parameters. The input to the die bond detection model is die bond images, and the output rep-resents the bonding status of the die’s sides and corners. In our previously published work [3], these were expressed in binary form as Good or Bad [16], as shown in Figure A5.

In our previously published work [3], Table A1 shows the die bond types corre-sponding to different combinations of hexadecimal codes, where s represents the die side bond code, c represents the die corner bond code, g indicates a well-bonded state (bond_good), and b indicates a poorly bonded state (bond_bad). Based on the hexadecimal codes corresponding to the model outputs, this study can statistically analyze the die bond status of all dies, identifying the locations where bonding defects most frequently occur, typically concentrated on specific sides or corners. It provides substantial practical value for automated quality monitoring and process optimization in semiconductor production lines.

**Figure A6:**
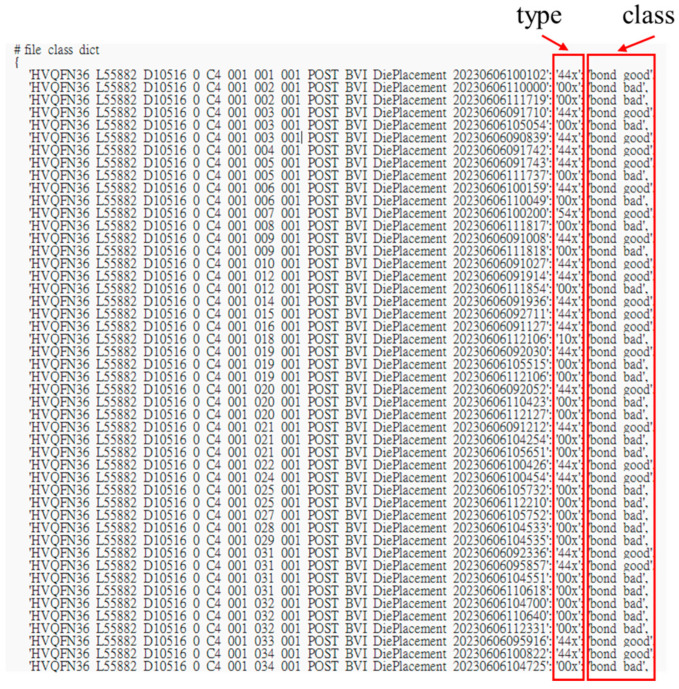
Prediction classification with the type of die bond.

In our previously published work [3], Figure A6 shows that during the testing phase, a separate folder for subsequent analysis can store the predicted classification results of the model. The model’s predictions then combine the actual classification re-sults of the test set to construct a confusion matrix for calculating precision and other performance metrics. Through the confusion matrix, we can intuitively observe the model’s classification performance across the four categories—side_good, side_bad, corner_good, and corner_bad—allowing a quick assessment of the model’s strengths and weaknesses. When the machine observes an increase in poorly bonded dies, the machine can promptly adjust the process parameters to prevent excessive production of defective dies. This approach not only enhances the reliability of detection but also helps the wafer fab reduce manufacturing costs, further ensuring the stability and yield of the die bond process.

### 2.5. CGSE-Yolov7-Tiny Model

**Figure 1:**
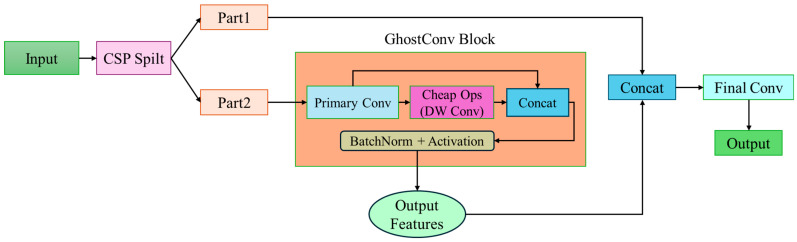
CSPGhostConv architecture.

Introducing Ghost Convolution (GhostConv) [17] significantly improves inference efficiency, enabling deep learning models to maintain high accuracy and stability while achieving lightweight characteristics. Figure 1 illustrates the CSPGhostConv architecture [18], which integrates the Cross Stage Partial (CSP) structure [19] with the GhostConv module. This architecture is a lightweight yet practical feature fusion module commonly employed to replace conventional convolutional layers, thereby accelerating inference and reducing the number of parameters.

People treated the CSPGhostConv module as a lightweight variant inspired by the CSP structure, in which the system further incorporates GhostConv to enhance computational efficiency and feature fusion capabilities. This modification makes it particularly suitable for deep learning architectures sensitive to inference speed.

The mechanism divides the workflow of this architecture into two main components. First, the direct path allows a portion of the input feature maps—typically 50% of the input channels—to bypass the main computation module through a skip connection and be directly concatenated at the final stage. This design preserves the original feature information and minimizes information loss. Second, the processing path processes the remaining portion of the input feature maps—typically 50% of the input channels—through the main computational module (e.g., GhostConv), where this architecture can perform complex feature transformations to learn new feature representations.

The Squeeze-and-Excitation (SE) Layer [20] constitutes a lightweight component engineered to enhance the performance of convolutional neural networks (CNNs). It enhances important features and suppresses less important ones by allowing the network to learn inter-channel relationships and dynamically recalibrate each channel, thereby improving model accuracy.

The SE Layer comprises two key steps: Squeeze and Excitation. Specifically, the Squeeze phase compresses the global spatial information from every channel to a representative value. The most commonly used method is Global Average Pooling (GAP). For an input feature map of size H × W × C, global average pooling compresses it into a 1 × 1 × C feature vector. Each value in this vector represents the average response of the corresponding channel across the spatial dimensions, capturing the global contextual information of that channel. Excitation: This step aims to learn a weight for each channel. The 1 × 1 × C feature vector obtained from the Squeeze step is fed into a fully connected layer with ReLU activation, reducing the number of channels to C/r, where r is the reduction ratio, reducing the number of parameters. This structure is followed by another fully connected layer that restores the channel number to C, using a Sigmoid function to constrain the output between 0 and 1. Essentially, these values represent each channel’s “excitation” or weighting factor.

**Figure 2:**
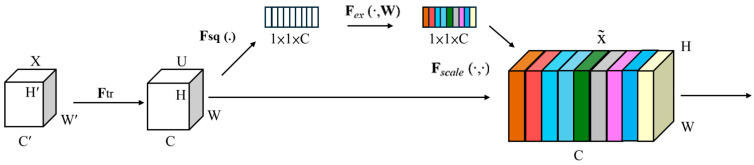
Squeeze-and-Excitation (SE-Net) module.

As the final step, this configuration uses the learned channel weights to recalibrate the features dynamically by applying them to the original input feature map through channel-wise multiplication, as depicted in Figure 2.

**Figure 3:**
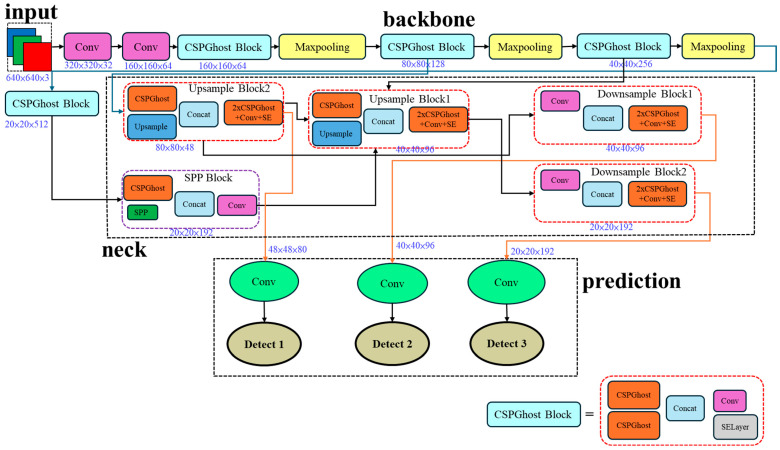
CGSE-Yolov7-tiny architecture.

Considering that GhostConv simulates high-cost convolutions through cheap operations, saving parameters and computations, and that CSP divides the input into two parts to reduce computation, these two modules were combined. Although this CSPGhostConv does not fully follow the CSPNet approach of splitting paths and merging, it mimics the spirit of “dual paths → concatenation → compression”. Combining the two outputs and then compressing helps maintain a balance between representational capability and lightweight design. Furthermore, this model can also utilize a Squeeze-and-Excitation (SE) Layer to enhance model performance, as shown in Figure 3.

### 2.6. Mobile-YOLOv7-Tiny Model

MobileNetV3 [21,22] is the next-generation MobileNet series model developed by Google, designed as an efficient convolutional neural network (CNN) specifically for mobile devices. It combines complementary search techniques and novel architectural designs aimed at optimizing for mobile CPUs, achieving a better balance between accuracy, latency, and model size. The development of MobileNetV3 utilized a hardware-aware neural architecture search (NAS) method, which considers specific hardware constraints, such as CPU performance, to reduce inference latency. In addition, it integrates the NetAdapt algorithm to optimize the model further, ensuring high accuracy under specific latency constraints. Based on NAS and NetAdapt, manual architectural improvements were made to enhance model efficiency, such as adjustments to bottleneck structures and nonlinear activation functions.

NAS is an automated search method for neural network architectures that simultaneously considers hardware performance constraints (e.g., latency, power consumption, memory usage). NAS strategies generally include Reinforcement Learning-based NAS, Evolutionary NAS, Gradient-based NAS (e.g., DARTS), and others. In MobileNetV3, the system uses Platform-aware NAS (MnasNet) to design the block combination sequence, aiming for accurate and fast networks on specific hardware. A recurrent neural network (RNN)-based controller and a factorized hierarchical search space were employed, with the reinforcement learning controller determining each block’s expansion ratio (e.g., 3, 6), kernel size (e.g., 3 × 3, 5 × 5), whether to use the SE module, and the activation function (ReLU or h-swish).

**Figure 4:**
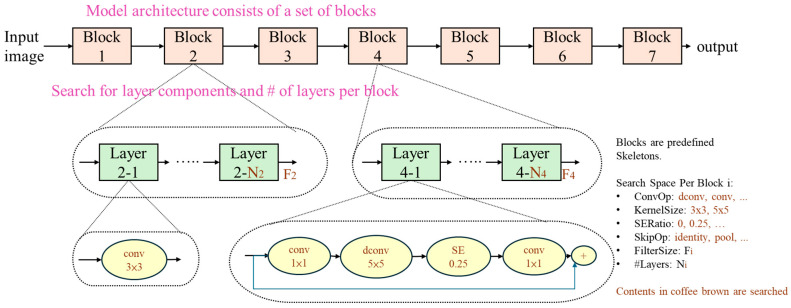
Hierarchical Search Space architecture.

In MNASNet (Mobile Neural Architecture Search Network), the factorized hierarchical search space divides the model into blocks and layers, addressing insufficient layer diversity. During the search, for each block, it determines “which operation each layer performs” and “how many times to repeat it.” The composition of layers can vary completely between different blocks. Figure 4 shows block 2 consists of standard convolution layers, while block 4 comprises bottleneck layers combined with squeeze-and-excitation layers.

The objective function proposed by MNasNet considers real-world application scenarios. Since MNasNet incorporates the target platform into the objective function, it is called platform-aware NAS. MNasNet aims to maximize this objective function.

(1) $$Objective\;Function=ACC\left(m\right)\times {\left[\frac{LAT(m)}{TAR}\right]}^{w}$$

**Figure 5:**
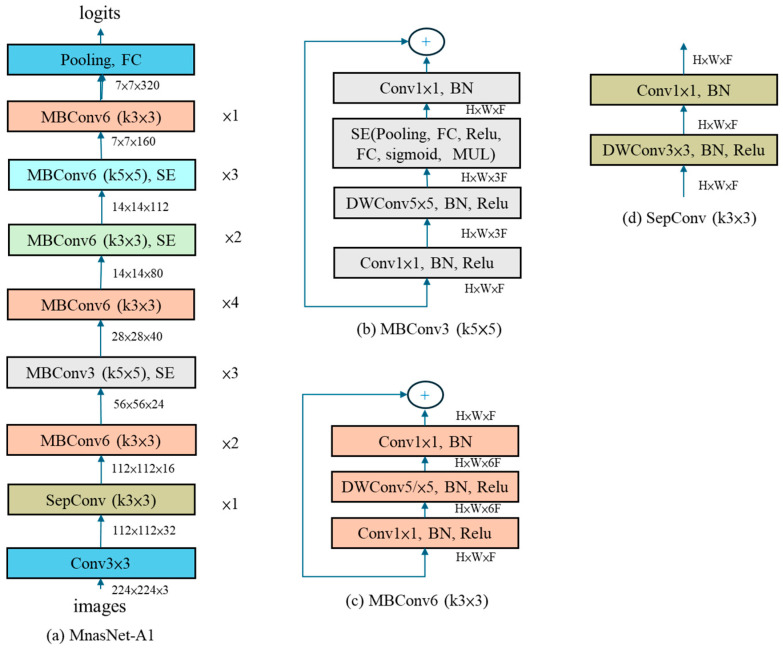
MnasNet-A1 network architecture.

Equation (1) represents the objective function, where ACC and LAT denote accuracy and latency, respectively, m represents the sampled model, TAR is the target latency, and w is an application-specific constant (considered a weight factor). In the original paper [21], we set w to −0.07. In MobileNetV3, the authors observed that the accuracy of lightweight models changes more sharply with latency, so they adjusted w to −0.15. Finally, the model obtained using this method is similar to the results reported in the MnasNet paper; therefore, the authors directly adopted MnasNet-A1 as the initial model for subsequent improvements, as shown in Figure 5.

**Figure 6:**
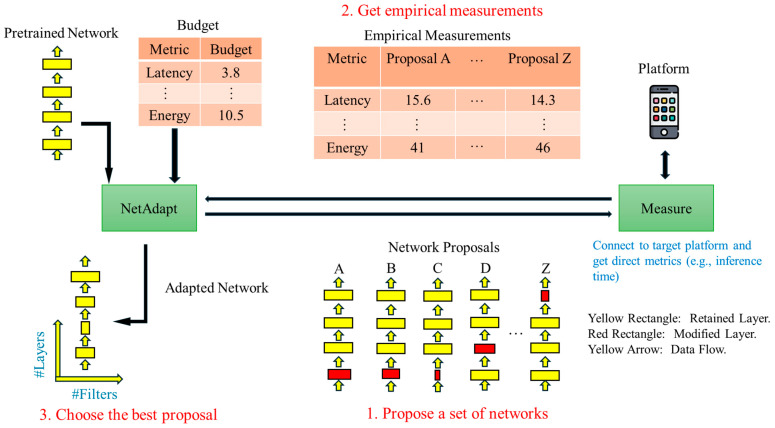
NetAdapt architecture.

The NetAdapt algorithm first generates a set of proposals, which modify the model to reduce its computation time by a specific amount. It employs each proposal to adjust the pre-trained model and fine-tune it to estimate its accuracy roughly. This algorithm then selects the best proposal according to specific criteria. This process repeats until it reaches the target computation time, as shown in Figure 6.

MobileNetV3 considers two types of proposals: reducing the size of the expansion layers and the size of the bottlenecks uniformly across all blocks. MobileNetV3 uses the change in accuracy relative to latency (accuracy/|latency|) as the criterion for model selection, choosing the proposal that minimally decreases accuracy while satisfying the first step. After identifying the final proposal, NetAdapt re-trains the model from scratch to obtain the final model.

**Figure 7:**
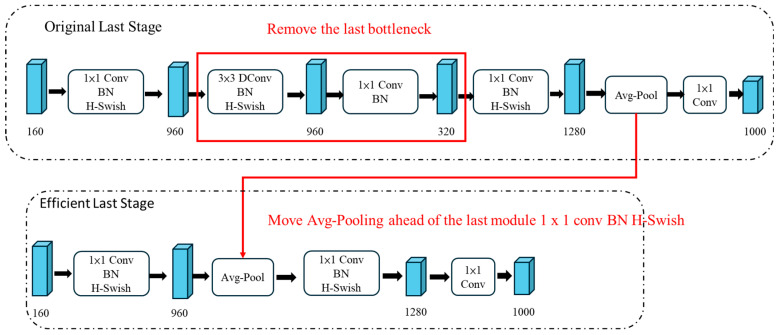
Improving MobileNetV2 (upper one) to the MobileNetV3 (lower one).

In the previous generation MobileNetV2, the final 1 × 1 convolution expanded the activation tensor into a higher-dimensional space to facilitate subsequent predictions. However, this also required a large amount of computation. MobileNetV3 moves this 1 × 1 convolution after the global average pooling, reducing the input size from 7 × 7 to 1 × 1, thereby decreasing the computational load. Because this change already reduces a substantial amount of computation, this improvement can remove the bottleneck blocks previously used for dimensionality reduction and information propagation, as shown in Figure 7.

Equation (2) represents the Swish function, a smooth and differentiable nonlinear activation function, where x is the input variable and σ(x) is the sigmoid function. Swish possesses a self-gated property, allowing it to adaptively adjust its output based on the input value, enhancing gradient flow and feature representation capability.

(2) $$Swish\left(x\right)=x\times \sigma \left(x\right)=x\times \frac{1}{1+e^{-x}}$$

MobileNetV3 uses ReLU6 to replace the sigmoid function, naming this function hard swish (h-swish). Equation (3) defines the h−swish function, which is a piecewise function with three segments: when the input x≤−3, the output is 0; when −3<x<3, the function follows a quadratic form x(x+3)/6; when x≥3, the output equals the input x itself. It is computationally simple, and ReLU6 can implement it efficiently. Within the range [−3,3], it approximates Swish. Since it avoids the expensive sigmoid, it is beneficial in low-latency models, offering performance similar to Swish while enjoying broader platform support for ReLU6.

Equation (4) defines the ReLU6 function, an improved version of the Rectified Linear Unit (ReLU). ReLU6 retains ReLU’s sparsity property while capping the output at 6, preventing numerical overflow during low-precision computations or quantization. This design enhances model stability and quantizability on mobile and embedded devices. By replacing sigmoid with ReLU6, h-swish avoids precision loss during quantization and can be represented as a piecewise function, reducing memory requirements on hardware. Combining linear and sigmoid characteristics, h-swish approaches zero as x→−∞ and x as x→+∞, remaining continuous and differentiable, making it suitable for deep networks.

(3) $$h-swish\left(x\right)=x\times \frac{ReLU6(x+3)}{6}$$

(4) $$ReLU6(x)=min(\max\left(0,x\right),6)$$

**Figure 8:**
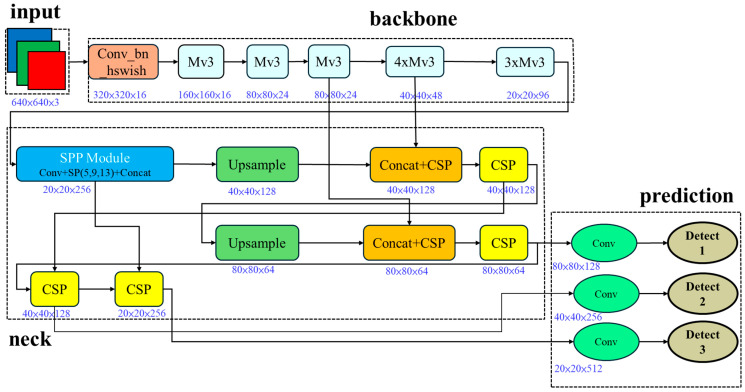
Mobile-YOLOv7-tiny architecture.

Compared to setting the number of SE channels based on the bottleneck size in MnasNet, MobileNetV3 fixes the number of SE channels to one-fourth of the expansion layer channels. MobileNetV3 uses channels of the expansion layer /4 to find the intermediate channels for the SE block to achieve the best performance. Figure 8 shows the overall architecture.

### 2.7. ReG-Yolov7-Tiny Model

**Figure 9:**
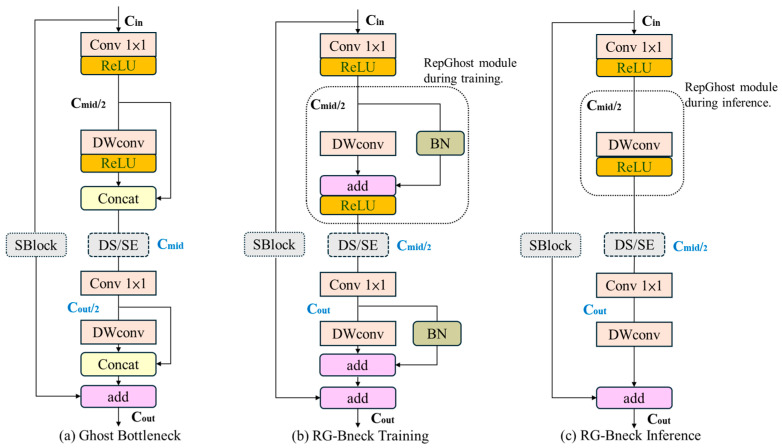
RepGhost architecture.

The RepGhost module [23] is a lightweight convolutional design that combines the Ghost module with reparameterization techniques, as shown in Figure 9. (a) A block from the GhostNet network. (b) A block of the RepGhost network during training. (c) A block of the RepGhost network is present during inference.

The core idea is that the input channels $C_{in}$ are first compressed within the bottleneck structure through a 1 × 1 convolution, reducing the input dimension. The model uses the intermediate channels $C_{mid}$ to decrease computational cost. Then, another 1 × 1 convolution restores the feature channels $C_{out}$ to the output dimension, forming a typical “shrink–expand” structure. Since depthwise convolution (DWConv) can only maintain a one-to-one correspondence between input and output channels without changing the channel number, it performs channel compression and expansion within the bottleneck using standard convolutions.

In the Ghost Bottleneck, intermediate feature extraction uses DWConv to generate redundant features, which are then concatenated with the primary convolution results to achieve efficient feature representation. Furthermore, in the RG-bneck module, a re-parameterization structure is introduced into the compressed DWConv during training to enhance feature representation; during inference, this structure can be folded into a single convolution, avoiding extra inference overhead, thus achieving a “training-enhanced, inference-efficient” design.

**Figure 10:**
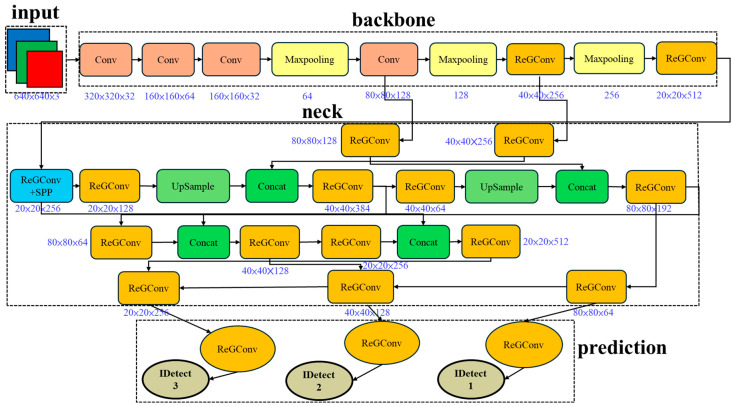
ReG-Yolov7-tiny architecture.

Additionally, the module incorporates a Shortcut Block (SBlock) that provides a residual connection path. When enabled, the input features can be directly added to the output, further improving gradient flow and convergence stability. Compared with the original Ghost module, RepGhost maintains efficient redundant feature generation while further strengthening model expressiveness and reducing structural complexity during inference, making it particularly suitable for deployment in resource-constrained scenarios, as shown in Figure 10.

### 2.8. DSGReG-Yolov7-Tiny Model

Depthwise Separable Convolution (DS Conv) [17] reduces computational load and decreases network parameters, which helps mitigate overfitting and improves inference speed. This function enables the model to balance lightweight design and high efficiency while maintaining accuracy.

Based on this design principle, previous studies [24,25] combines DS Conv with Ghost Convolution to form a simplified convolutional module called DSG Conv. Using DSG Conv, the network can significantly reduce computational cost and model parameters while preserving feature representation capability, achieving lightweight and fast inference in convolutional neural networks. In practical applications, previous studies [24,25] proposed replacing some convolutional YOLOv7-tiny with DSG Conv layers to lighten the model architecture. This modification retains the network’s ability to extract complex features and effectively enhances inference speed and computational efficiency.

**Figure 11:**
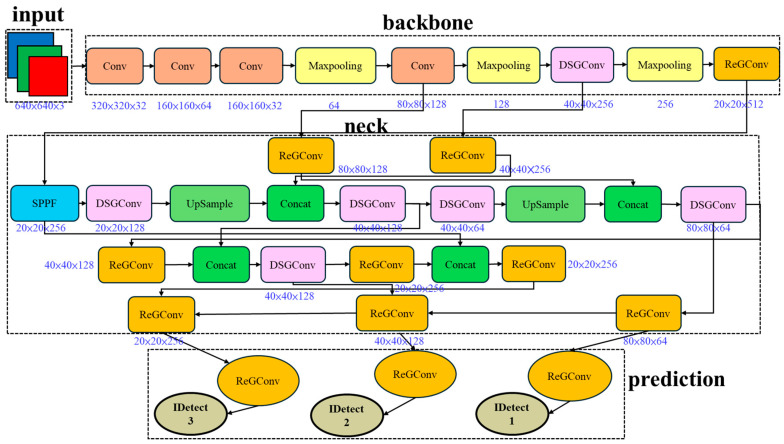
DSGReG-Yolov7-tiny architecture.

The Depthwise Ghost Convolution leverages depthwise separable convolution combined with Ghost feature generation to efficiently reduce computation and parameters while maintaining feature representation. Re-parameterization introduces multi-branch structures during training to enhance model expressiveness and collapses them into a single path during inference, drastically reducing computational overhead. These techniques enable a highly accurate, low-latency, and efficient model suitable for deployment in resource-constrained environments, as illustrated in Figure 11.

### 2.9. DSGβSI-Yolov7-Tiny Model

As noted previously, while Leaky ReLU partially mitigates ReLU’s limitations by allowing small negative outputs, its fixed negative slope often fails to adapt to diverse data distributions or network layer characteristics optimally. In practical applications, the negative outputs introduced by Leaky ReLU may sometimes affect the network’s convergence behavior and learning capacity, resulting in performance degradation. Previous studies [15,26] have indicated that properly improving the design of activation functions can further enhance inference accuracy and model stability. Based on this insight, this study replaces the original Leaky ReLU activation function in the DSG-YOLOv7-tiny architecture with the Sigmoid Linear Unit (SiLU).

SiLU is an activation function with smooth characteristics, effectively alleviating the jitter problem during training and enabling more stable gradient updates. Compared with ReLU and Leaky ReLU, SiLU exhibits a soft activation behavior between linear and nonlinear transformations. This activation allows it to capture subtle variations in the input data more flexibly while maintaining better gradient stability throughout the training process. Such properties enhance the model’s ability to represent complex features and improve its generalization capability. As validated by the subsequent experimental results, integrating SiLU into DSG-YOLOv7-tiny yields a slight yet consistent improvement in precision and accuracy, demonstrating its practical value in improving model performance.

Like Leaky ReLU, SiLU produces negative outputs for negative inputs, thereby retaining a non-zero gradient in the negative domain. Equation (5) defines the Sigmoid function, where x denotes the input and σ(x) represents the output. When x approaches positive infinity, $e^{-x}$ approaches zero, and σ(x) approaches 1. Conversely, when x approaches negative infinity, $e^{-x}$ grows very large, and σ(x) approaches 0. Thus, the Sigmoid function maps any real-valued input x to the interval [0,1], making it widely applicable in probability interpretation and nonlinear modeling.

Building on this, Equation (6) defines the SiLU function, where x is the input and σ(x) is the Sigmoid function described above. By combining a linear term with the smooth nonlinear modulation of Sigmoid, SiLU maintains an approximately linear response in the positive domain, while smoothly preserving a small portion of negative outputs in the negative domain. This design avoids the complete inactivation problem of traditional ReLU in the negative region. Due to its balance between smoothness and nonlinearity, SiLU ensures more stable gradient propagation, facilitating faster model convergence and improving overall prediction accuracy.

(5) $$\sigma (x)=1/(1+e^{-x})$$

(6) silu(x)=x·σ(x)

In some cases, using SiLU has resulted in only marginal improvements in accuracy. Consequently, two SiLU-based variants—Modified SiLU and Adaptive SiLU—have been proposed to address this limitation. The motivation behind adopting these modified activation functions is to maintain a high level of accuracy while pursuing improved inference speed.

Equation (7) defines the Modified SiLU function, where x represents the input, MoSiLU(x) denotes the output, and β is a learnable parameter that controls the steepness of the activation curve. When the input x is large, σ(βx) approaches 1; conversely, when x is small, σ(βx) approaches 0. Initially, β is set to 1.0, resulting in a fixed curve slope and limited adaptability to different feature levels across the network.

To address this limitation, β is progressively adjusted layer by layer. Specifically, it is first increased to 1.5 to enhance the expression capability of nonlinear features, then gradually reduced to 1.25 to create a smoother curve. This gradual modulation facilitates more stable gradient flow, improves the learning efficiency of different layers, and ultimately enhances inference accuracy.

(7) Mosilu(x)=x·σ(βx)

Equation (8) describes the formulation of MoSiLU, where activation uses x to compute the adaptive scaling factor, and $\beta_{a}(x)$ represents a small neural network. The weight matrix $W_{1}$ of the first layer and the weight matrix $W_{2}$ of the output layer of $\beta_{a}(x)$ are both randomly initialized, while $b_{1}$ and $b_{2}$ are biases initialized to zero. This structure functions similarly to the learning behavior of a fixed β, but provides greater flexibility by dynamically adjusting the activation strength according to the input signal.

Furthermore, Equation (9) presents the Adaptive SiLU function, where x denotes the input and b is the bias, initialized to −1. When b=−1, the function behaves similarly to ReLU around x=0, resulting in $\sigma (\beta_{a}(x)\cdot x+b)\approx 0$, which preserves nonlinear characteristics while adapting to scale variations of different inputs. This design allows the activation function to be input-sensitive and to capture diverse feature representations across different network layers more effectively.

(8) $$\beta_{a}\left(x\right)=W_{2}\cdot relu(W_{1}x+b_{1})+b_{2}$$

(9) $$Apsilu(x)=x\cdot \sigma (\beta_{a}\left(x\right)\cdot x+b)$$

According to the Xavier initialization method [27,28], Equation (10) defines the initialization of the weight matrices $W_{1}$ and $W_{2}$. Equation randomly drew the weight values from a uniform distribution U(a,b). Here, $n_{in}$ represents the input dimension of the layer (in this study, the input image size is 1024 × 1024), and $n_{out}$ stands for the output dimension (corresponding to the number of classes, which is 4 in this case). The coefficient $\sqrt{6}$ serves as the Xavier initialization factor, which helps maintain stable weight variance during forward propagation.

After initialization, the training updated the weights through backpropagation. The network performs a forward pass to generate predictions and calculate the loss. Then, it computes the loss gradient with respect to the weights. Finally, an optimizer (such as gradient descent) updates $W_{1}$ and $W_{2}$ in the direction that minimizes the error. The training iterates this process continuously, allowing the model to improve its performance gradually.

In this structure, the input dimension of $W_{1}$ corresponds to the previous layer’s output, and its output dimension corresponds to the number of neurons in the hidden layer. The input dimension of $W_{2}$ corresponds to the hidden layer output, and its output dimension is 1, representing the adaptive scaling factor $\beta_{a}(x)$.

(10) $$W∼U(-\frac{\sqrt{6}}{\sqrt{\left(n_{i}+n_{out}\right)}},\;\frac{\sqrt{6}}{\sqrt{\left(n_{i}+n_{out}\right)}})$$

**Figure 12:**
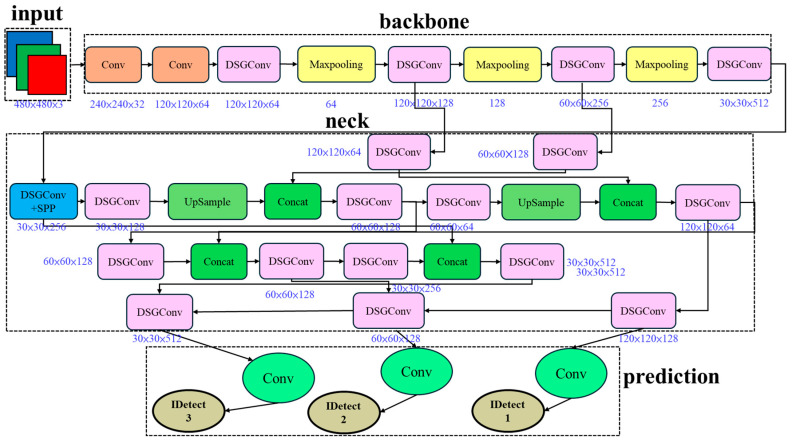
DSGβSI-Yolov7-tiny architecture.

As illustrated in Figure 12, the DSGβSI-YOLOv7-tiny architecture first introduces modifications to the backbone. When β is at 1.0, the slope of the activation curve becomes inflexible, limiting the function’s ability to adapt. To address this issue, the activation employs Modified SiLU in the early layers (Layers 0, 1, 5, and 10), where β is initially set to 1.5 and then reduced to 1.25 after two layers. This adjustment improves gradient flow in the early stages of the network.

In the later layers of the backbone, the original activation retains SiLU to ensure gradient stability and maintain a balance between feature extraction and information propagation. Subsequently, the neck section adopts Adaptive SiLU, enabling the network to better handle the complex feature combinations in intermediate stages. Finally, Adaptive SiLU is also applied in the head section, allowing the model to dynamically adapt and select the most suitable activation behavior for accurate prediction.

## 3. Method

The methodology of this chapter begins with data preparation and preprocessing, followed by model selection and parameter configuration, and then proceeds to model training. After training, various metrics and visualizations can evaluate the models’ performance, such as confusion matrices, precision-recall (PR) curves, and loss plots, to assess whether the trained model has achieved the training objectives.

Subsequently, the trained model tests the models on die bond image recognition, where the test classifies the die bonds as bond_good or bond_bad. Finally, we will compare each model’s performance based on relevant evaluation metrics, allowing for an assessment of the relative effectiveness of the different models.

### 3.1. Model Enhancement

Although the previously proposed DSGβSI-YOLOv7-tiny model effectively improved performance through the enhanced activation function, its convolutional layers still employ relatively low-complexity structural configurations, making it difficult to surpass the 99% accuracy threshold. To further improve the model’s feature representation capability and detection accuracy, this study incorporates the previously described Squeeze-and-Excitation (SE) Layer into the architecture, as shown in Figure 2.

The core concept of the SE Layer involves a “Squeeze” operation that performs global average pooling to compress channel information, followed by an “Excitation” mechanism that adaptively reweights channel importance. This design strengthens key features while suppressing redundant information, significantly improving the model’s feature selection ability and generalization performance with minimal additional parameters and computational cost.

Within the DSGβSI-YOLOv7-tiny architecture, the SE Layer is inserted into selected critical convolutional modules, enhancing the representation of informative channels during feature extraction. By focusing the network on features highly relevant to the target detection task and reducing sensitivity to background noise, this module improves the accuracy and stability of detection results. Notably, the SE Layer has a limited impact on computational complexity and inference speed, enabling the model to maintain high-speed inference while achieving higher accuracy and better generalization.

**Figure 13:**
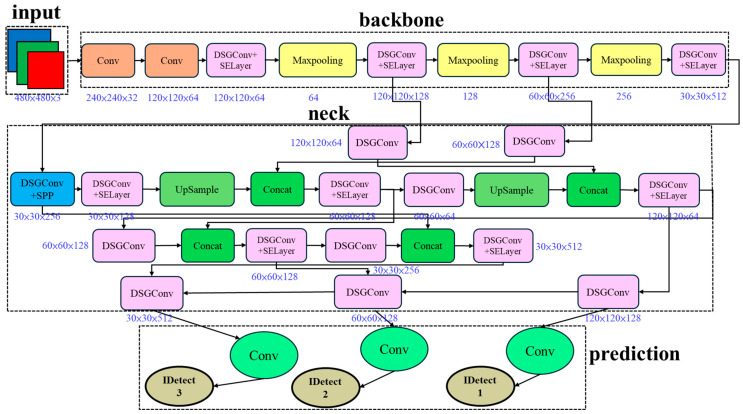
DSGβSI-SE-Yolov7-tiny architecture.

Experimental results demonstrate that the combination of DSGβSI activation functions and SE Layer allows the model to efficiently balance accuracy and efficiency when handling complex die bond image inspection tasks. As shown in Figure 13, this further validates the practicality and advantages of the proposed methodology.

### 3.2. Improving Lightweight Model

To further enhance model performance, particularly in inference speed, the model should be faster than DSGβSI-YOLOv7-tiny. However, improving speed often comes at the cost of reduced accuracy, which represents a key challenge in model design. To address this issue, this study performs architectural fine-tuning based on DSGβSI-YOLOv7-tiny by modifying specific modules. These improvements aim to enhance the model’s accuracy in small-object detection while compensating for the relative limitations of lightweight models in feature extraction and attention mechanisms.

To address these issues, this study proposes a multi-attention fusion design, integrating channel attention, comprising SE-Layer [20] and Efficient Channel Attention (ECA) Net [29], with spatial-channel joint attention (Coordinate Attention) to enhance the model’s capture of critical features. Coordinate Attention preserves precise positional information, enhancing the network’s spatial awareness and localization capability. Simultaneously, this study introduces the Small Object Enhancer as a dedicated strategy to improve the feature representation for small objects and the performance of lightweight models when processing small targets. This approach effectively improves detection performance and accuracy while maintaining the lightweight nature of the model.

The DSGβSI-SECS-YOLOv7-tiny architecture is a fine-tuned version of DSGβSI-YOLOv7-tiny, incorporating the SECS module, which consists of four components: SE Layer, ECA net, Coordinate Attention, and Small Object Enhancer (collectively abbreviated as SECS). SECS simultaneously attends to feature information from multiple perspectives, including channel relationships, spatial positions, and coordinate information. The SE Layer models inter-channel dependencies via a global receptive field and uses adaptive weighting to recalibrate channel importance, thereby enhancing feature representation dynamically.

### 3.3. Enhancing the Network’s Representation and Reducing the Computation in Excitation

The Squeeze-and-Excitation Layer (SE-Layer) [20] addresses the problem of unequal importance across channels by adaptively recalibrating channel weights, highlighting essential channels. That is, the SE Layer adaptively weights the channel features of the original dimensions to enhance useful features and suppress useless features, thereby improving the network’s representation ability and generalization performance. However, SE Layers tend to have larger parameter counts and higher computational complexity, which motivates the adoption of ECA (Efficient Channel Attention) to replace the part of excitation in the SE-layer. Equation (11) computes the weight of feature maps to increase the weight of important feature maps and decreasing the weight of unimportant feature maps, where z represents the input feature map, s stands for the channel weight vector, $W_{1}$ is a fully connected layer weight matrix, passing through a ReLU denoted δ, $W_{2}$ is a fully connected layer weight matrix, going through a sigmoid function indicated σ, W implies a linear transformation matrix, and $F_{ex}$ takes a mapping function for the execution block. The ECA module replaces fully connected layers with 1D convolutions, effectively reducing parameter count and improving computational efficiency.

(11) $$s=F_{ex}\left(z,W\right)=\sigma \left(g\left(z,W\right)\right)=\sigma (W_{2}\delta (W_{1}z))$$

**Figure 14:**
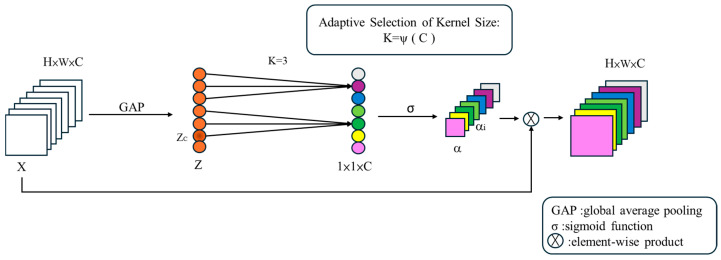
ECA-Net architecture.

ECA [29] adaptively weights channel-wise features to enhance informative and suppress irrelevant ones, improving network representation and generalization. The advantage of ECA is that it replaces the fully connected hierarchical FC linear transformation and nonlinear function mapping, as shown in Equation (11), used in the traditional SE layer to calculate the excitation. By implementing channel attention via 1D convolution, ECA achieves a lightweight design, maintaining performance while reducing computational cost. As illustrated in Figure 14, the input tensor first undergoes Global Average Pooling (GAP), followed by a 1D convolution of size k, and finally, an element-wise product with the input tensor produces the output. Here, k is adaptively determined based on the channel dimension C.

Lightweight models inherently have restricted feature extraction and attention modeling capabilities, constraining their representational power. Furthermore, conventional attention mechanisms often overlook spatial positional encoding, further limiting performance in localization tasks. Despite these improvements, several challenges remain in designing lightweight models. For instance, the modified DSGβSI-YOLOv7-tiny still exhibits limited accuracy in small-object detection, indicating that detecting small targets remains difficult.

### 3.4. Adding Spatial Position and Coordination Information

**Figure 15:**
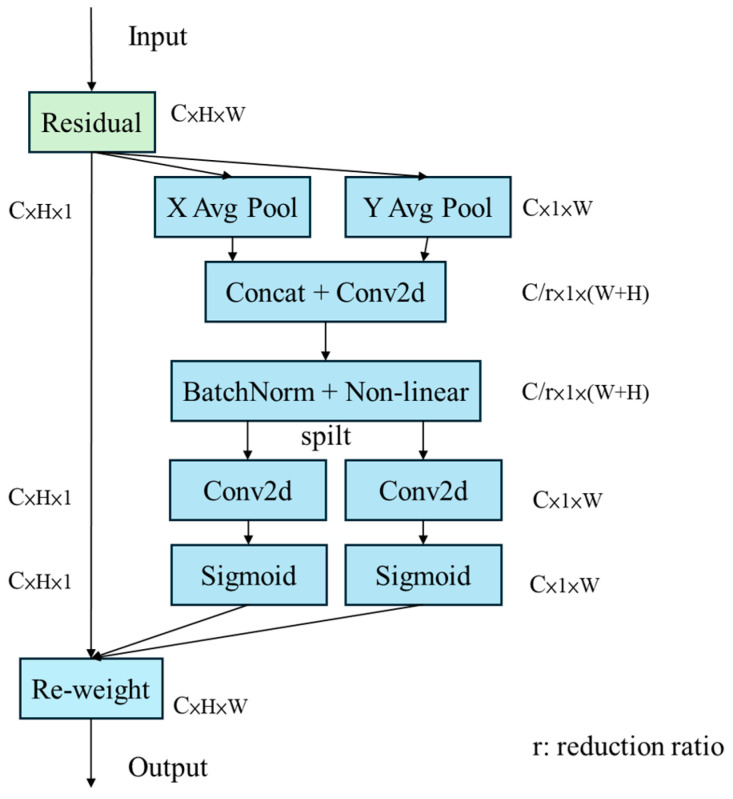
Coordinate Attention workflow.

The Coordinate Attention (CA) module [30] addresses the limitation of traditional attention mechanisms that often lose spatial positional information by embedding positional cues into channel attention, thereby capturing both spatial orientation and location-sensitive features. Figure 15 illustrates the operational workflow of Coordinate Attention. First is to take the feature map output from a given model layer as input within the CA module. Global average pooling is then applied along the height and width dimensions separately to extract key features in the vertical and horizontal directions.

Subsequently, the features from both directions are fused and processed through compression and nonlinear activation operations to reduce the number of parameters while enhancing feature representation. Then, the module splits into two independent attention branches, restores the number of channels, and takes activations corresponding to the vertical and horizontal directions, which capture crucial positional information along the height and width. Finally, these attention maps are applied to the input feature map to reweight and enhance important regions, improving the model’s object localization and feature learning capabilities.

In the Coordinate Attention module, the reduction ratio parameter r plays a critical role in controlling the channel dimensionality reduction. Specifically, CA performs global average pooling along the horizontal and vertical directions of the input feature map, producing two sets of direction-aware feature descriptors. These descriptors are concatenated along the channel dimension and fed into a 1 × 1 convolution layer for channel compression. The reduction factor r determines the degree of this dimensionality reduction. An appropriately chosen r preserves sufficient semantic and directional information while reducing parameter count and computational complexity. If r is too large, excessive compression may impair the model’s ability to capture inter-channel dependencies. Conversely, if r is too small, additional computational overhead is introduced, reducing inference efficiency.

### 3.5. Improving Small Object Detection

To address the information loss issue for small objects within the feature pyramid, this study develops the Small Object Enhancer (SOE) module to strengthen feature representation for small targets and improve the retention and utilization of fine-grained features. A key advantage of this module is its four-branch parallel processing design, which avoids inter-branch dependencies and fully leverages the parallel computation capabilities of GPUs.

Regarding information preservation, all convolution operations employ appropriate padding to maintain spatial dimensions. They use Residual connections to retain the original feature information, preventing information loss. At the adaptive enhancement level, the network learns to integrate features across different scales and automatically adjusts feature weights for various small objects, thereby enhancing detection performance. Moreover, the module is computationally efficient: it first performs dimensionality reduction to reduce computational load, followed by standard convolution operations, making the overall structure hardware-friendly and suitable for acceleration.

**Figure 16:**
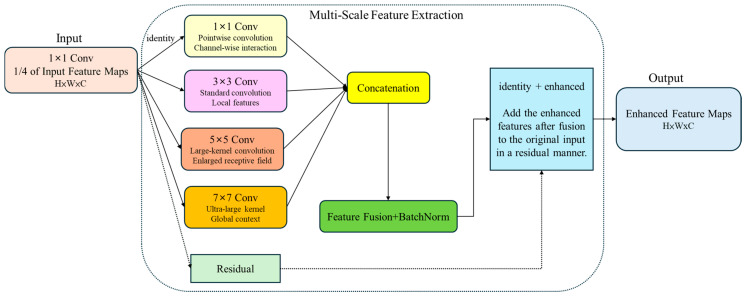
Small Object Enhancer workflow.

Figure 16 illustrates the operational workflow of the Small Object Enhancer. The Small Object Enhancer module reduces the input feature channels to one-fourth using a 1 × 1 convolution, lowering computational cost while preserving critical information. It then applies 1 × 1, 3 × 3, 5 × 5, and 7 × 7 convolutions to extract detailed information at multiple receptive fields [31]. The module concatenates and fuses these multi-scale features along the channel dimension. Finally, it adds the fused features to the original input via a residual connection, enhancing the representation of small objects and maintaining gradient stability.

### 3.6. Further Lightweight Model

**Figure 17:**
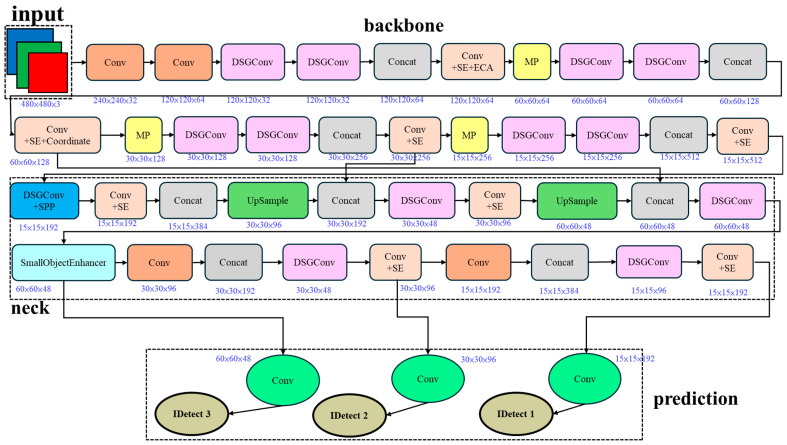
DSGβSI-SECS-Yolov7-tiny architecture.

Modifying specific DSGConv modules in DSGβSI-YOLOv7-tiny and integrating the SE Layer, ECA net, Coordinate Attention, and Small Object Enhancer allows the newly proposed model to feature information from multiple perspectives and levels simultaneously. Specifically, it captures inter-channel relationships, spatial positions, and coordinate information, compensating for previous lightweight models’ limitations. This design enables a more efficient and intelligent detection approach, as illustrated in Figure 17.

### 3.7. Model Building

This study trained a total of seven models for the die bond inspection task, including CGSE-YOLOv7-tiny, Mobile-YOLOv7-tiny, ReG-YOLOv7-tiny, DSGReG-YOLOv7-tiny, DSGβSI-YOLOv7-tiny, DSGβSI-SE-YOLOv7-tiny, and DSGβSI-SECS-YOLOv7-tiny. For each model, parameters were initially randomly initialized and then iteratively optimized through trial-and-error during training to achieve the best-performing configurations, as summarized in Table 1. Table 1 presents the optimal parameter combinations for each model in this study.

**Figure 18:**
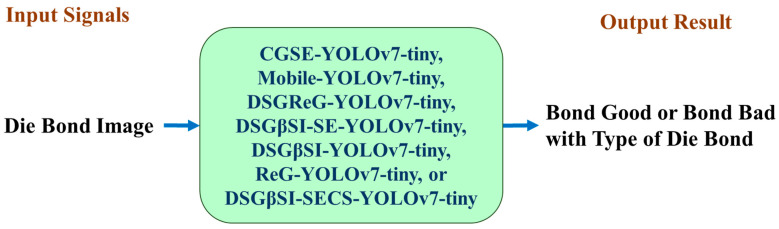
Detection and prediction model using Yolo-related models.

Figure 18 illustrates the detection workflow, where the model processes an input image to generate predictions of die bond status. The model can distinguish between bond_good and bond_bad targets and identify the die bond type, indicating the adhesion status along the four edges and corners of the die.

### 3.8. Workflow of the System

This study used 3145 images provided by a semiconductor manufacturer for data preprocessing. The preprocessing workflow included organizing the image dataset and annotating each image using LabelImg, converting the original XML labels into the format required by YOLO. Subsequently, the dataset was split into training, validation, and test sets, with proportions of 70%, 15%, and 15%, respectively.

The training set the number of epochs to 300, the batch size to 32, and resized all input images to 1024 × 1024 pixels. The selected models for training included CGSE-YOLOv7-tiny, Mobile-YOLOv7-tiny, ReG-YOLOv7-tiny, DSGReG-YOLOv7-tiny, DSGβSI-YOLOv7-tiny, DSGβSI-SE-YOLOv7-tiny, and DSGβSI-SECS-YOLOv7-tiny.

**Figure 19:**
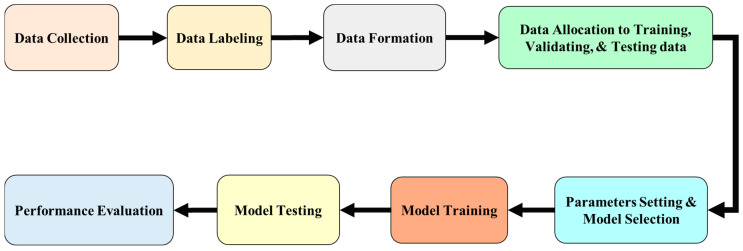
Workflow diagram.

After training, this experiment performed model inference and recognition on the test set, and recorded the performance of each model during both training and testing phases to evaluate and compare their effectiveness, as shown in Figure 19.

## 4. Experimental Results and Discussion

The goal of this experiment is to verify whether the proposed approach, which utilizes image sensing and object detection, can achieve the expected results. The experimental steps included collecting and labeling sensor images, retraining the model, testing it, and finally comparing the proposed approach with several benchmark methods on various performance metrics.

### 4.1. Experiment Setting

Table 2 lists the hardware environment used in this study, while Table 3 summarizes the software configuration. During the data preprocessing stage, we first annotated each image using LabelImg. The annotation files were then converted from XML format to text files using Jupyter Notebook, splitting the dataset into training, validation, and test sets.

Subsequently, model training and inference were performed in an Anaconda Prompt environment using Python and PyTorch, with TensorBoard employed to monitor the training process. Based on the inference results, Jupyter Notebook classified dies into bond_good (fully bonded) and bond_bad (partially bonded or defective) categories.

Furthermore, the experiment evaluated the performance of multiple models, and the best-performing model was exported in TensorFlow Lite format via Anaconda Prompt, making it suitable for deployment in production line machines.

### 4.2. Data Collection and Model Evaluation

The experiments were conducted primarily in an Anaconda 3 environment. Initially, the experiment trained the models CGSE-YOLOv7-tiny, Mobile-YOLOv7-tiny, ReG-YOLOv7-tiny, DSGReG-YOLOv7-tiny, DSGβSI-YOLOv7-tiny, DSGβSI-SE-YOLOv7-tiny, and DSGβSI-SECS-YOLOv7-tiny on a PC workstation and subsequently deployed them on a Jetson Nano embedded platform to compare the performance of different models. We deployed the improved models to the Jetson Nano for performance evaluation.

The dataset acquired from a high-resolution image-sensing camera comprised 3145 images from a well-known semiconductor manufacturer in southern Taiwan. Dataset 1, collected by Machine #1, included 2491 images used for training, while Dataset 2, collected by Machine #2, contained 474 images used for testing. This study designed a cross-dataset setup to evaluate the models’ generalization ability.

Each sensed image was labeled for annotation using LabelImg, followed by standard data preprocessing. The experiment split the training dataset into 2204 images (≈70%) for training and 467 images (≈15%) for validation. We tested object detection models on the workstation platform and employed a cross-validation [32] strategy with k = 5 during training and validation to comprehensively assess the YOLO series models’ performance.

The study also recorded the training time for each model using the same training dataset on the workstation. Equation (12) calculates the total inference time $IT_{i}$ for each model on the 474 test images, where i denotes the i-th object detection model, I is the total number of models, x represents the x-th test image, X is the total number of test images, and $EIT_{i}$ Indicates the time required for the model to complete inference on a single image.

(12) $$IT_{i}=\sum_{x=1}^{X}EIT_{i},\;\;where\;\;i=1,\;2,\;\ldots ,I,\;\;x=1,\;2,\ldots ,\;X$$

In the experimental setup, we set the test image size to 1024 × 1024 pixels, the batch size was 32, and the number of epochs was 300. Table 4 presents the results, where the first row shows the training time required for each object detection model under the same parameter settings, and the second row reports the inference time for completing 474 test images.

The experimental results indicate that the proposed DSGβSI-SECS-YOLOv7-tiny outperforms the other models regarding inference time, demonstrating superior computational efficiency.

Table 5 compares the evaluated object detection models’ parameter counts and FLOPs (Gflops). Before applying DSGConv for model lightweighting, CGSE-YOLOv7-tiny had the most significant parameters, followed by Mobile-YOLOv7-tiny, whose parameter count remained relatively high for a lightweight target. In contrast, the DSGβSI-SECS-YOLOv7-tiny model exhibited the smallest parameter count, demonstrating the most effective lightweight performance.

Moreover, ReG-YOLOv7-tiny and its subsequent improved versions, including DSGReG-YOLOv7-tiny, DSGβSI-YOLOv7-tiny, DSGβSI-SE-YOLOv7-tiny, and DSGβSI-SECS-YOLOv7-tiny, all achieved significant reductions in parameter counts compared to CGSE-YOLOv7-tiny and Mobile-YOLOv7-tiny. This result confirms that the proposed modifications successfully compressed the models while improving computational efficiency, meeting the intended design objectives for lightweight and high-performance object detection.

### 4.3. Experimental Results

**Figure 20:**
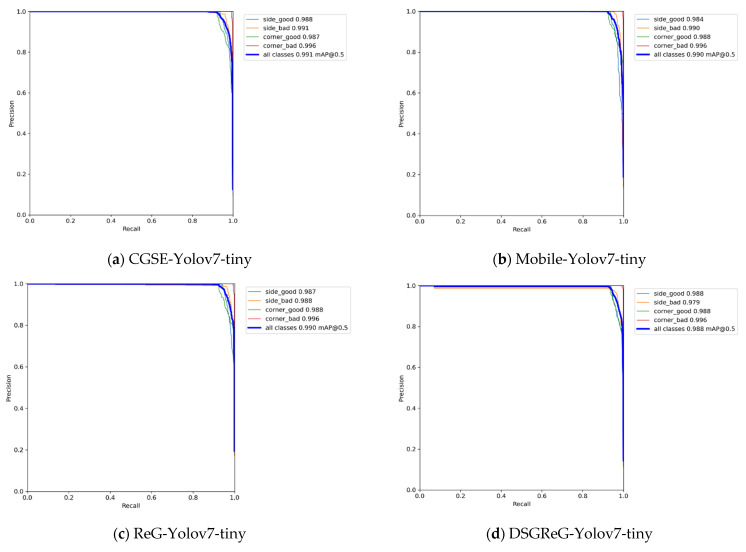

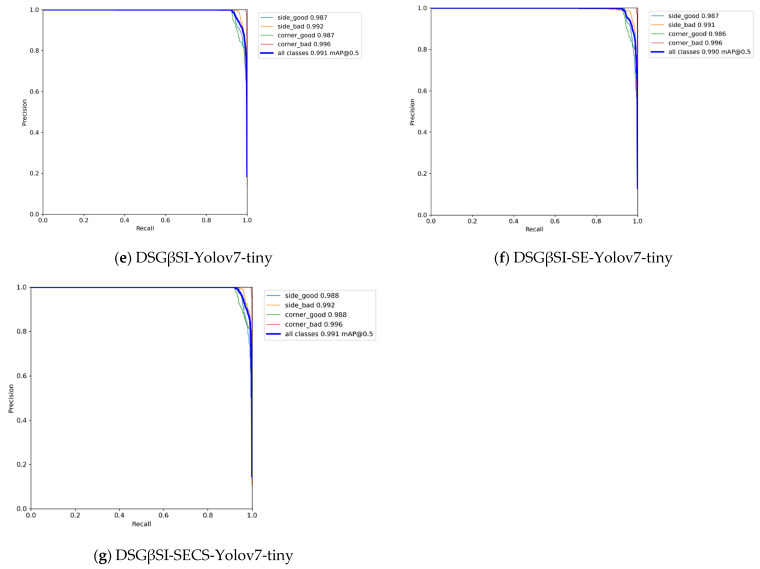
The precision-recall curve for the object detection model.

**Figure 21:**
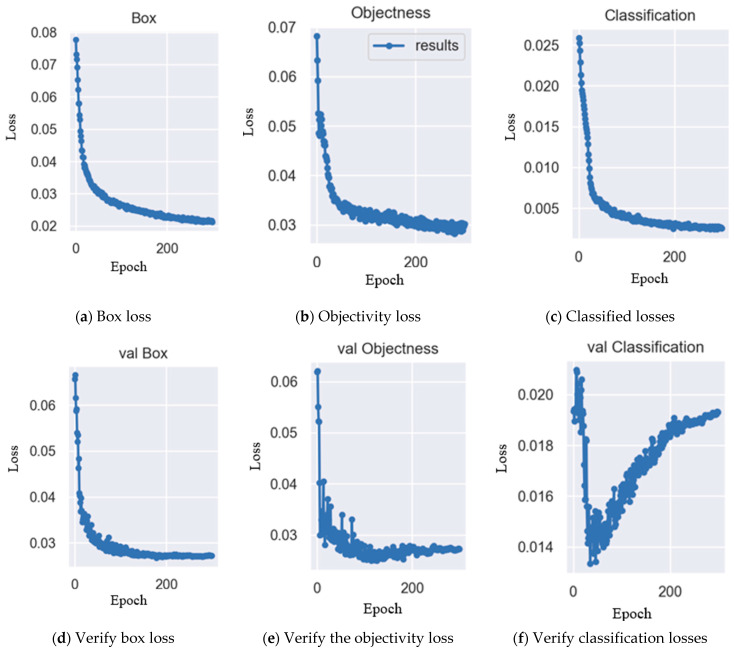
Loss plot of DSGβSI-SECS-Yolov7-tiny.

- In Figure 20, the PR (Precision-Recall) curves plot Recall on the X-axis and Precision on the Y-axis, with each point representing the Recall and Precision obtained at different threshold values. The mAP (mean Average Precision) is calculated by summing the AP (Average Precision) values for all classes derived from the PR curve and dividing by the total number of classes.
- The PR curves show that DSGβSI-YOLOv7-tiny exhibits the lowest precision, achieving only 98.7%. Meanwhile, CGSE-YOLOv7-tiny and Mobile-YOLOv7-tiny, despite having larger parameter scales, maintain at least 99% precision. Interestingly, DSGReG-YOLOv7-tiny, which applies DSGConv, does not outperform ReG-YOLOv7-tiny in terms of precision.
- Since the experiments primarily focused on the previously proposed DSGβSI-YOLOv7-tiny, we conducted subsequent model refinements. The improved DSGβSI-SE-YOLOv7-tiny showed a noticeable increase in precision. Furthermore, the proposed DSGβSI-SECS-YOLOv7-tiny, designed for more efficient and intelligent detection, achieved an accuracy of 99.1%, matching that of CGSE-YOLOv7-tiny, while offering a more computationally efficient model compared to DSGβSI-YOLOv7-tiny.
- After completing model training, this study further employed visualization tools to generate Loss plots to observe the convergence behavior during training. Under a batch size of 32, the experiment trained the DSGβSI-SECS-YOLOv7-tiny model for 300 epochs, and Figure 21 shows the resulting Loss plot.
- During the 300 epochs, the experiment generated six plots to illustrate the model’s training loss and convergence patterns. In Figure 21, the first, second, and third rows correspond to the Localization Loss, Confidence Loss, and Classification/Objectness Loss (representing the matching between predicted boxes and ground truth). Additionally, the first and second columns represent training loss and validation loss, where the abbreviation “val” in the second column indicates the validation set.

### 4.4. Performance Evaluation

- The execution performance of object detection models can be quantified using Equation (13) to calculate the Frames Per Second (FPS). According to this formula, each object detection model yields a corresponding $FPS_{j}$, where j denotes the j-th object detection model, and J represents the total number of models. ${IRAIT}_{j}$ refers to the time required for the j-th model to process a single image under real-time image input conditions.
- By comparing the $FPS_{j}$ values across different models, one can evaluate their execution speed and performance differences in real-time application scenarios.

(13) $$IT_{i}=\sum_{x=1}^{X}EIT_{i},\;\;where\;\;i=1,\;2,\;\ldots ,I,\;\;x=1,\;2,\ldots ,\;X$$

- This study adopts mean Average Precision (mAP) to evaluate object detection accuracy. The calculation involves first computing the Average Precision (AP) for each class and then taking the mean across all classes to obtain the overall mAP.
- Equation (14) calculates the mAP of different object detection models as ${mAP}_{l}$, where L represents the total number of object detection models, l denotes the l-th model for calculating mAP, and $C_{l}$ is the number of classes that the model must recognize. For each class $k_{l}$, ${AP}_{k_{l}}$ stands for the Average Precision of that specific class.
- Using this approach, the experiment can comprehensively compare the precision performance of different models in multi-class recognition tasks.

(14) $$mAP_{l}=\frac{\sum_{k_{l}=1}^{C_{l}}{AP}_{k_{l}}}{C_{l}},\;\;where\;k_{l}=1,\;2,\;\ldots ,\;C_{l},\;l=1,2,\ldots ,L$$

- We compared the performance of different object detection models regarding inference speed and accuracy. This experiment trained all models under the same parameter settings and evaluated them on the same set of 474 test images, with corresponding Precision-Recall (PR) curves plotted and output. The inference speed (FPS) and accuracy (mAP) for each model were calculated according to Equation (13) and Equation (14), respectively. Table 6 summarizes the results.
- Table 6 compares multiple YOLO variants, including CGSE-YOLOv7-tiny, Mobile-YOLOv7-tiny, ReG-YOLOv7-tiny, DSGReG-YOLOv7-tiny, DSGβSI-YOLOv7-tiny, DSGβSI-SE-YOLOv7-tiny, and DSGβSI-SECS-YOLOv7-tiny, in various performance metrics, including FPS, Precision, Recall, F1-score, and Accuracy. The experimental results indicate that DSGβSI-SECS-YOLOv7-tiny outperforms the other models across all evaluated metrics, demonstrating superior overall performance.

### 4.5. Discussion

- Table 6 compares the performance metrics of various YOLO-series models, with the proposed DSGβSI-SECS-YOLOv7-tiny model emerging as the fastest by achieving an inference speed of 294.1 FPS. Compared to our previously published DSGβSI-YOLOv7-tiny [3], the inference speed in this study increased by 8.4 frames per second. The slowest model was CGSE-YOLOv7-tiny, reaching only 172.4 FPS, indicating room for improvement in speed performance.
- In terms of classification accuracy, DSGβSI-YOLOv7-tiny had the lowest precision among all models at 98.7%, while Mobile-YOLOv7-tiny and CGSE-YOLOv7-tiny, despite having slower inference speeds, achieved at least 99.0% precision. By applying the SE Layer to DSGβSI-YOLOv7-tiny, the resulting DSGβSI-SE-YOLOv7-tiny increased precision by 0.3% and accuracy by 0.5%, although its FPS decreased by 7.9 frames, failing to meet the target for lightweight design.
- To address this, the internal architecture of DSGβSI-YOLOv7-tiny was further modified by incorporating SE Layer, ECA net, Coordinate Attention, and Small Object Enhancer modules, resulting in the DSGβSI-SECS-YOLOv7-tiny model. This model demonstrated a clear advantage in inference speed, increasing FPS by 8.4 frames compared to DSGβSI-YOLOv7-tiny, while precision improved from 98.7% to 99.1%, surpassing that of DSGβSI-SE-YOLOv7-tiny. Therefore, DSGβSI-SECS-YOLOv7-tiny offers a balanced combination of high-speed computation and high accuracy, making it the most efficient object detection model in this study.
- Previous studies have informed the design and optimization of these models. Recognizing the constraints of limited memory and computational resources on embedded devices, Gu et al. [12] proposed using depthwise separable convolutions (DWConv) instead of standard convolutions in ELAN blocks to lower parameters and FLOPs. Gong et al. [14] and Tang et al. [15] also emphasized that small-scale networks such as YOLOv7-tiny and GhostNet can replace conventional convolutions to reduce computational burden. Based on these insights, this study removed redundant blocks from the original YOLOv7-tiny architecture and introduced optimized convolution layers to reduce computation while maintaining reasonable predictive accuracy significantly.
- Inspired by MobileNetV3 research from Howard et al. [21] and Hua et al. [22], the design principles were applied to form Mobile-YOLOv7-tiny, showing further potential for inference time improvement. Moreover, the advantages of combining YOLOv7-tiny with Ghost modules were highlighted by Gong et al. [14] and Tang et al. [15], while Oniga et al. [18] and Chen et al. [23] contributed CSP structure and reparameterization approaches, leading to the development of CGSE-YOLOv7-tiny, ReG-YOLOv7-tiny, and DSGReG-YOLOv7-tiny in this study.
- To enhance the performance of -YOLOv7-tiny, the SE module proposed by Zeng et al. [20] was adopted to recalibrate channel feature weights adaptively, emphasizing critical channels. The ECA module by Luo et al. [29] mitigated the additional parameter overhead of SE, and the Coordinate Attention module by Wang et al. [30] improved spatial awareness. The integration of these designs culminated in the DSGβSI -SECS-YOLOv7-tiny model, which offers faster inference and enhanced precision over prior models, making it highly suitable for real-time and high-efficiency die bond inspection applications.
- A limitation of this study arises when a single image contains multiple die bonds of different types. In such cases, the model may fail to learn the specific features for each bond type effectively during training, potentially compromising inference accuracy. An ideal solution would involve cropping multi-die images into single-die bond images for training and inference, which could improve performance but significantly increase preprocessing time. Additionally, although DSGβSI-SECS-YOLOv7-tiny performs exceptionally in inference speed, its precision is still 99.1%, and its accuracy is 98.5%. Pursuing even higher precision and accuracy may negatively impact inference speed.
- Furthermore, GPU performance is critical to precision, accuracy, and inference speed. This study used an NVIDIA GeForce RTX 4070 Ti. Supposing we can adopt an NVIDIA RTX 4090 with 24 GB GDDR6X memory or higher capability ones. By supporting larger-scale deep learning models, this embedded platform offers significant benefits for high-throughput, real-time die bond detection and prediction applications.

## 5. Conclusions

- This study focused on enhancing the original DSGβSI-YOLOv7-tiny image sensing and object detection model in terms of speed and accuracy. With the upgrading of automated production equipment and the emergence of more powerful image sensing and object detection methods, the proposed approach streamlines redundant convolutional modules in DSGβSI-YOLOv7-tiny, and incorporates modules—namely the SE Layer, ECA Net, Coordinate Attention, and Small Object Enhancer—designed to improve object detection. These improvements allowed the model to maintain high-speed inference while improving precision, enabling real-time and high-efficiency die bond detection.
- Moreover, we can rapidly deploy the proposed method onto factory production equipment to perform real-time classification for each die bond image and monitor the occurrence frequency of defective patterns. This method provides actionable feedback for adjusting critical machine parameters, enhancing production yield, and effectively reducing manufacturing costs. It improves production efficiency and significantly minimizes production losses for semiconductor production lines.
- People can extend the proposed method to broader application domains, including chip contour inspection, street scene analysis, operator personal protective equipment monitoring, and production line product inspection. In terms of performance enhancement, future work may involve further integrating more advanced YOLO variants, such as YOLOv13, while pursuing lightweight and optimized network architectures to improve object detection and image recognition capabilities.
- Regarding methodological improvements, future developments could explore multi-modal data fusion, edge computing deployment, adaptive reinforcement learning, data augmentation, and model compression and acceleration, to meet the stringent requirements of real-time performance and reliability in smart factories. Ultimately, the proposed approach advances die bond inspection and contributes significantly to Industry 4.0 smart manufacturing and high-precision image recognition applications.

## Figures and Tables

**Table 1:** Hyperparameters setting.

Model	Hyperparameters
CGSE-Yolov7-tiny	ep = 300, bs = 32, is = 1024 × 1024, op = AdamW, act = Leaky ReLU, Sigmoid, tc = $loss\leq 10^{-3}$
Mobile-Yolov7-tiny	ep = 300, bs = 32, is = 1024 × 1024, op = AdamW, act = ReLU6, h-swish, tc = $loss\leq 10^{-3}$
ReG-Yolov7-tiny	ep = 300, bs = 32, is = 1024 × 1024, op = AdamW, act = Leaky ReLU, Sigmoid, tc = $loss\leq 10^{-3}$
DSGReG-Yolov7-tiny	ep = 300, bs = 32, is = 1024 × 1024, op = AdamW, act = Leaky ReLU, Sigmoid, tc = $loss\leq 10^{-3}$
DSGβSI-Yolov7-tiny	ep = 300, bs = 32, is = 1024 × 1024, op = AdamW, act = ModifiedSiLU, AdaptiveSiLU, SiLU, Sigmoid, tc = $loss\leq 10^{-3}$
DSGβSI-SE-Yolov7-tiny	ep = 300, bs = 32, is = 1024 × 1024, op = AdamW, act = ModifiedSiLU, AdaptiveSiLU, SiLU, Sigmoid, tc = $loss\leq 10^{-3}$
DSGβSI-SECS-Yolov7-tiny	ep = 300, bs = 32, is = 1024 × 1024, op = AdamW, act = ModifiedSiLU, AdaptiveSiLU, SiLU, Sigmoid, tc = $loss\leq 10^{-3}$

p.s.: ep represents epoch, bs stands for batch size, is indicates image size, op means optimizer, ap denotes activation function, and tc is termination condition.

**Table 2:** Hardware Specifications.

Resource	Workstation
GPU	NVIDIA GeForce RTX 4070 Ti
CPU	Intel(R) Xeon(R) W-2223 CPU @ 3.60 GHz
Memory	Samsung DDR5 4800 32GB
Storage	Kingston NV3 1TB M.2 2280 NVMe SSD
Jetson Nano	NVIDIA Maxwell™ architecture with 128 NVIDIA CUDA^®^ cores

**Table 3:** Recipe of Packages.

Software	Version
LabelImg	1.8
Anaconda^®^ Individual Edition	4.9.2
Jupyter Notebook	6.1.4
TensorFlow	v2.14.0
PyTorch	1.6
Python	3.6.9

**Table 4:** Training and Inference Times.

Phase	CGSE-Yolov7-Tiny	Mobile-Yolov7-Tiny	ReG-Yolov7-Tiny	Dsgreg-Yolov7-Tiny	DSGβSI-Yolov7-Tiny	DSGβSI-SE-Yolov7-Tiny	DSGβSI-SECS-Yolov7-Tiny
Training (hr)	39.4	23.4	6.3	6.1	5.4	5.4	5.3
Inference (ms)	5.8	4.4	3.5	3.7	3.5	3.6	3.4

**Table 5:** Parameter and flop.

Feature	CGSE-Yolov7-Tiny	Mobile-Yolov7-Tiny	ReG-Yolov7-Tiny	DSGReG-Yolov7-Tiny	DSGβSI-Yolov7-Tiny	DSGβSI-SE-Yolov7-Tiny	DSGβSI-SECS-Yolov7-Tiny
Parameter (#)	4,818,322	3,830,090	744,658	767,443	743,829	754,698	742,552
Flop (Gflops)	11.9	6.6	1.9	2.2	1.8	2.1	1.7

**Table 6:** Performance indexes.

Metric	CGSE-Yolov7-Tiny	Mobile-Yolov7-Tiny	ReG-Yolov7-Tiny	DSGReG-Yolov7-Tiny	DSGβSI-Yolov7-Tiny	DSGβSI-SE-Yolov7-Tiny	DSGβSI-SECS-Yolov7-Tiny
FPS	172.4	227.3	285.7	270.3	285.7	277.8	294.1
Precision	99.1	99.0	99.0	98.8	98.7	99.0	99.1
Recall	95.3	95.2	95.4	95.3	95.3	95.3	95.3
F1-Score	0.97	0.97	0.97	0.97	0.97	0.97	0.97
Accuracy	98.5	99.0	98.5	97.5	98.5	99.0	98.5

Note: the unit of Precision, Recall, and Accuracy is percentage.

## Data Availability

The Sample Programs used to support the findings of this study can be found at the following link: https://drive.google.com/open?id=16ew1qH6AuyC-jq8UZbNCeM3e5irJANOT&usp=drive_fs (accessed on 16 October 2025).

## Appendix A

**Table A1:** The type of die bond.

	s	0	1	2	3	4	5	6	7	8	9	A	B	C	D	E	F
c																	
0		b	b	b	b	b	b	b	g	b	b	b	g	b	g	g	g
1		b	b	b	b	b	b	b	g	b	b	b	g	b	g	g	g
2		b	b	b	b	b	b	b	g	b	b	b	g	b	g	g	g
3		b	b	b	b	b	b	b	g	b	b	b	g	b	g	g	g
4		b	b	b	b	b	b	b	g	b	b	b	g	b	g	g	g
5		b	b	b	b	b	b	b	g	b	b	b	g	b	g	g	g
6		b	b	b	b	b	b	b	g	b	b	b	g	b	g	g	g
7		g	g	g	g	g	g	g	g	g	g	g	g	g	g	g	g
8		b	b	b	b	b	b	b	g	b	b	b	g	b	g	g	g
9		b	b	b	b	b	b	b	g	b	b	b	g	b	g	g	g
A		b	b	b	b	b	b	b	g	b	b	b	g	b	g	g	g
B		g	g	g	g	g	g	g	g	g	g	g	g	g	g	g	g
C		b	b	b	b	b	b	b	g	b	b	b	g	b	g	g	g
D		g	g	g	g	g	g	g	g	g	g	g	g	g	g	g	g
E		g	g	g	g	g	g	g	g	g	g	g	g	g	g	g	g
F		g	g	g	g	g	g	g	g	g	g	g	g	g	g	g	g

P.S.: The symbol “b” represents bad, and the symbol “g” stands for good.
